# Exploring the intersection of sustainable consumption and the Metaverse: A review of current literature and future research directions

**DOI:** 10.1016/j.heliyon.2023.e19190

**Published:** 2023-08-17

**Authors:** Alfonso Pellegrino, Ray Wang, Alessandro Stasi

**Affiliations:** a Sasin School of Management, Chulalongkorn University, Sasa Patasala Building Soi Chula 12, Phyathai Road, Bangkok 10330, Thailand; b Thammasat University, 99 Moo 18 Paholyothin Road, Klong Luang, Rangsit, Prathumthani 12121 Thailand; c Mahidol University International College, 999 Phutthamonthon Sai 4 Rd, Salaya, Phutthamonthon District, Nakhon Pathom 73170, Thailand

**Keywords:** Review, Metaverse, Consumption

## Abstract

This study aims to analyze the intersection between the Metaverse and sustainable consumption by examining and reviewing 21 journal articles and to identify future research directions for studies in this domain. The review identifies five domains of literature in the field, namely smart cities, social sustainability, tourism, education, and sustainable marketing. Our findings suggest that these areas offer valuable insights for sustainable consumption in the Metaverse. The authors highlight the need for a comprehensive understanding of the economic and environmental impacts of the Metaverse on sustainable consumption. Finally, the study identifies future research directions, including a better understanding of consumer behavior, policy considerations, motivations in the Metaverse and the role of technology in promoting sustainable consumption. This study contributes to the emerging field of Metaverse and sustainable consumption by providing an overview of the current domains of literature in the field.

## 1 Introduction

With the growing integration of digital technology in everyday life, the advent of virtual environments, particularly the Metaverse, signifies an important shift in our societal dynamics [1]. The Metaverse, a term derived from science fiction, has transpired into a tangible reality due to recent advances in artificial intelligence and immersive technologies such as virtual and augmented reality [2]. The Metaverse, more than just a mere virtual space, symbolizes a fusion of digital environments that use augmented reality (AR) and virtual reality (VR), establishing a universally accessible collective virtual shared space. Here, digital avatars can engage in diverse activities, ranging from interaction, work, shopping, attending classes, to participating in social gatherings [3].

The Metaverse is often viewed as a potential arena for sustainable development, posing a novel intersection between digital transformation and sustainable consumption. However, studies dissecting the specific sustainability challenges and potential solutions within the Metaverse remain sparse. Hence, this paper aims to bridge this gap in literature, offering a comprehensive examination of the existing literature on the interplay between the Metaverse and sustainable consumption [4].

The current review defines sustainability in the Metaverse as the degree to which it addresses economic, environmental, and societal issues, with an emphasis on sustainable consumption. Through an analysis of 21 journal articles sourced from established academic databases - Scopus and ScienceDirect, domains of literature such as smart cities, social sustainability, tourism, education, and sustainable marketing are identified and analyzed [3,5].

Upon identifying the gaps in current literature, this study primarily seeks to presents a valuable overview of the impacts of the Metaverse on sustainable consumption. To achieve this, the study delves into the intricate aspects of consumer behavior in the Metaverse, examining motivations and the role of technology in promoting sustainable consumption [6]. We propose that a deepened comprehension of these areas is pivotal for fostering sustainable practices in the Metaverse, thereby contributing to the larger goal of a sustainable future.

Our contribution to the current body of literature is threefold:

1. We deepen the understanding of the Metaverse's role in sustainable consumption, delving into the mechanisms and potential for digital platforms to drive sustainability.
2. We illuminate the gaps and future research opportunities at the juncture of the Metaverse and sustainable consumption, underscoring under-explored areas that are ripe for investigation.
3. We present an in-depth analysis of the Metaverse's potential in addressing economic, environmental, and societal issues, examining the implications of digital technologies for sustainable development.

Our overarching objective is to elucidate the role of the Metaverse in sustainable consumption, identify existing knowledge gaps, and examine the potential of digital platforms in promoting sustainability. We scrutinize this through a detailed literature review and a subsequent comprehensive analysis of the Metaverse's potential to tackle economic, environmental, and societal challenges.

The article is organized as per the outline: Section two offers a detailed review of the current literature, focusing on the Metaverse and sustainable consumption. Section three discusses the methodology and results of our review and addresses the reviewers' concerns about the scientific quality of the paper. Lastly, we discuss the implications of our findings and future research areas, concluding with the contributions and practical implications of our study.

## 2 Background overview

The Metaverse has recently taken center stage as a growing virtual environment, gaining significant traction in contemporary discourse [7,8]. This renewed interest was notably sparked by Facebook's rebranding to ‘Meta’. Subsequently, numerous industry leaders, including Microsoft, Apple, and Roblox, have expressed a growing interest for the Metaverse. The present iteration of the Metaverse intertwines real-life through virtual currency, potentially fostering a deeper sense of social meaning beyond existing online technologies [8].

The Metaverse and consumption present a complex interrelation. The existing literature can be divided and categorized according to three primary themes: 1) Environmental impacts, 2) Economic impacts, and 3) Social and ethical implications.

1. **Environmental impacts:** Studies have conjectured that the Metaverse holds potential to reduce carbon emissions, as digital replacements for physical goods become more prevalent and physical activities like mobility and construction are reduced [9,10]. However, the specific extent to which this substitution can alleviate environmental concerns remains understudied, presenting a knowledge gap in understanding the real-world impact of digital replacements [11].
2. **Economic impacts:** Nevertheless, technology equipment and larger data networks do contribute to energy consumption and CO2 emissions. Cryptocurrencies, an integral part of the Metaverse, rely on energy-intensive blockchain technology, while cloud computing data centers require significant electricity to cool the servers [12]. The move towards less energy-intensive technologies is promising, but there is a lack of rigorous assessment of the net sustainability impact of such changes, indicating another area for further exploration [13].
3. **Social and ethical implications:** As outlined by recent literature, the Metaverse could contribute to social and ethical sustainability [14,15]. While some tech companies are prioritizing sustainability objectives, the transparency, stability, and sustainability of these systems are under-investigated. This points to a critical gap in establishing robust ethical standards and protocols within the Metaverse [16].

In addition to these, there are two more themes connected with the Metaverse and sustainable consumption:

4. **Sustainable marketing:** The Metaverse can transform marketing strategies and promote sustainable consumption [17]. However, there is a lack of comprehensive understanding of how to most effectively leverage the Metaverse for sustainable marketing practices, thus presenting another research opportunity [18].
5. **Smart cities:** The Metaverse can contribute to the creation of smart cities, enhancing the sustainability of urban environment [11]. But the specific mechanisms of how the Metaverse can facilitate such urban sustainability are not yet fully understood, signaling another knowledge gap.

To address the identified gaps and better comprehend the potential sustainability consumption issues within the Metaverse, it is crucial to understand its implications for sustainability, encompassing the environmental, social, and ethical impacts. Building upon the work by Jauhiainen et al. [15], our study further extends the understanding of the sustainability dimensions of the Metaverse, relating it to the specific aspect of sustainable consumption. By identifying the domains of literature in the field, we aim to explore potential research avenues to contribute to a more sustainable future.

## 3 Methods

### 3.1 Search strategy and selection criteria

The study followed the Preferred Reporting Items for Systematic Reviews and Meta-Analyses (PRISMA) guidelines [19]. The PRISMA flow diagram, illustrating our search strategy, selection process, inclusion and exclusion criteria, is included in the manuscript.

Our systematic review started with keyword searches aimed to encompass the breadth of the topic. The initial keyword searches are included in Table 1

**Table 1:** Keywords combination.

Combination No.	Keyword 1	Boolean Operator	Keyword 2	Boolean Operator	Keyword 3	No. of Articles Found
1	Metaverse	AND	Sustainab*			59
2	Metaverse	AND	Sustainab*	AND	Consumption	4
3	Metaverse	AND	Virtual Reality	AND	Sustainab*	25
4	Metaverse	AND	Augmented Reality	AND	Sustainab*	7
5	Metaverse	AND	Mixed Reality	AND	Sustainab*	5

We decided to focus on the search string: “Metaverse” AND “sustainab*”, as it produced the most relevant results for our research (see Table 2, Table 3, Table 4, Table 5). Also, the authors realized that by adding the keyword “consumption” the number of paper was too limited to carry out a significant review and we ran the risk of not including papers who discussed the interrelation between Metaverse and sustainable consumption not in a direct way.

**Table 2:** Identification of documents.

	Identified from Database	Included in the Review
ScienceDirect	21	4
Scopus	38	17
**Total**		21

**Table 3:** Identification of advantages and disadvantages about the Metaverse.

Pros	Cons
Improved resource management in urban centers	Economic gaps may not be solved and could worsen urban inequality
Reduced testing and modeling costs through digital twins technology	Inclusivity may be limited due to the cost of hardware, potentially perpetuating social segregation
Potential to reduce energy consumption and carbon emissions through decreased travel	Ethics, privacy, and security issues may arise, hindering adoption
Enhanced social interactions and inclusivity	Blind and deaf people may not be able to interact in the Metaverse
Tourism benefits such as phygital experiences, preservation of heritage sites, and increased financial resources for tourism-related businesses	Some physical activities cannot be replicated in the virtual environment
Potential for improved urban planning, transit, energy generation, health, education, entertainment, and other dimensions affecting quality of life.	Serious concerns with issues such as surveillance, marginalization, and dystopianism, requiring explicit process and practices for enhancing public participation and allowing democracy to shape and control the development of the Metaverse.

**Table 4:** Most highly cited documents.

No.	Authors	Documents	Citations
1	Choi H.-Y. et al. (2022)	Building Korean DMZ Metaverse Using a Web-Based Metaverse Platform	31
2	Golf-Papez M. et al. (2022)	Embracing falsity through the Metaverse: The case of synthetic customer experiences	26
3	Zhou Y. et al. (2022)	Self-powered sensing technologies for human Metaverse interfacing	26
4	Arpaci I. et al. (2022)	Understanding the social sustainability of the Metaverse by integrating UTAUT2 and big five personality traits: A hybrid SEM-ANN approach	18
5	Lee H.J. et al. (2022)	Technology-Enhanced Education through VR-Making and Metaverse-Linking to Foster Teacher Readiness and Sustainable Learning	12
6	Valaskova K. et al. (2022)	Virtual Marketplace Dynamics Data, Spatial Analytics, and Customer Engagement Tools in a Real-Time Interoperable Decentralized Metaverse	7
7	Allam Z. et al. (2022)	The Metaverse as a Virtual Form of Smart Cities: Opportunities and Challenges for Environmental, Economic, and Social Sustainability in Urban Futures	6
8	Park S. et al. (2022)	Identifying World Types to Deliver Gameful Experiences for Sustainable Learning in the Metaverse	6
9	Garrido-Iñigo P. et al. (2015)	The reality of virtual worlds: pros and cons of their application to foreign language teaching	5
10	Cheung J.C.-W et al. (2022)	Virtual reality based multiple life skill training for intellectual disability: A multicenter randomized controlled trial	5

**Table 5:** Identification of advantages and disadvantages about the Metaverse's implications.

Pros	Cons
The Metaverse offers a new platform for government officials to experience and predict the impact of their decisions on the environment.	The current iterations of the Metaverse have caused physical ailments such as dizziness and headaches, and it may not be viable for the Metaverse to require headsets.
Virtual consumption in the Metaverse has the potential to decrease the need for physical relocation, reducing carbon footprints and providing access to a wider variety of products and services without the need for physical travel.	The shift towards virtual consumption may lead to a decrease in the demand for physical goods, which could have unintended consequences for industries that rely on the manufacture and sale of physical products.
Virtual consumption in the Metaverse can provide a more immersive and interactive learning experience, which can lead to improved retention of information and a decrease in the need for physical textbooks, reducing the environmental impact of the educational sector.	The increased use of technology for education in virtual environments may lead to an increase in energy consumption and carbon emissions, and the unequal distribution of access to technology may create disparities in access to education.
Virtual environments in the Metaverse provide a unique opportunity for companies to engage with their target audience in a more immersive and interactive way, leading to increased brand awareness and a deeper connection with customers, and can help to reduce the environmental impact of traditional marketing methods.	Virtual consumption in the Metaverse may contribute to overconsumption and a culture of disposability, and virtual goods and experiences require energy and resources to produce, even if they do not have a physical form.
The Metaverse can reduce the need for physical travel, which can have a significant impact on urban sustainability, and provide a platform for urban planners to test new green infrastructure and sustainability initiatives.	Virtual environments can contribute to the sprawl of urban development and the consumption of resources and energy, increasing the overall consumption of resources and energy and contributing to the degradation of the environment.
Virtual consumption in the Metaverse has the potential to drive sustainable consumption patterns, but it is important to consider the potential consequences for the environment, and the policies which must be adopted to guarantee that virtual environments are equitable and sustainable.	The current literature lacks clear objectives related to the Metaverse, and the issue of technology addiction, sexual harassment, and bullying should be addressed.

### 3.2 Databases and document types

The databases we used for the systematic literature review were Science Direct and Scopus, and these choices were based on their wide coverage of disciplines relevant to our study (including information technology, social science, and sustainability). For transparency, we tracked and reported the number of articles found in these databases.

We considered different types of documents in our search, including journal articles, conference papers, and book chapters. However, we only included peer-reviewed journal articles in our review that were relevant to the topic we wanted to explore.

### 3.3 Selection criteria and process

Detailed inclusion and exclusion criteria were developed to guide the selection of papers. The primary inclusion criteria were that papers had to describe the Metaverse related to sustainable consumption using both quantitative or qualitative research methods and include quantitative or qualitative empirical evaluation of the application. We excluded papers that did not meet these criteria, as well as those that were duplicates, not written in English, or focused solely on technical aspects of the Metaverse without discussing sustainability implications.

Our paper selection process was iterative. Initially, we scanned the titles and abstracts of all the selected papers. After the first screening, full-text articles were assessed for eligibility. Any disagreements were resolved through discussion among the review team.

We also used backward search in our strategy. We checked the reference lists of the included studies to identify additional potentially relevant articles, a process known as backward citation tracking.

**Fig. 1:**
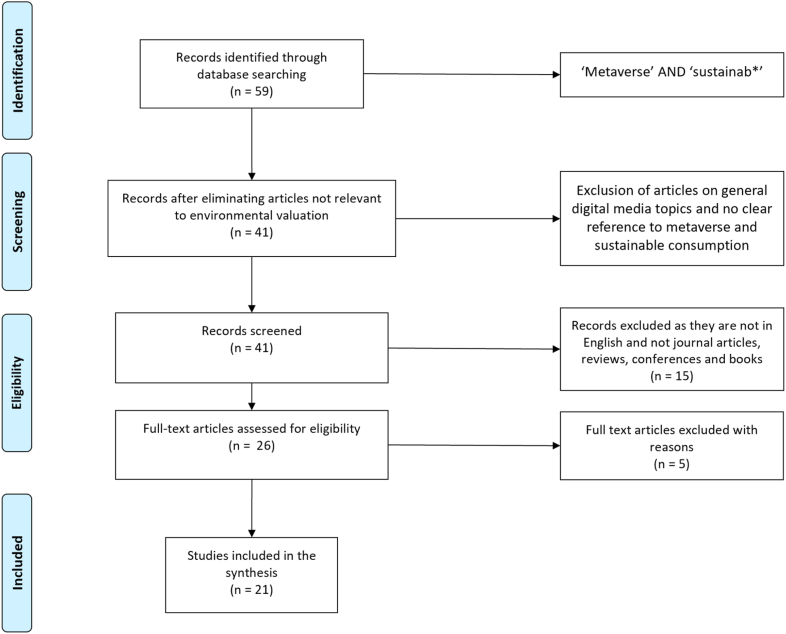
Prisma Diagram of articles' selection.

In total, our search yielded 59 articles published from 2015 to 2022, of which 21 met our selection criteria and were included in the final review. The final sample and selection process are detailed in Fig. 1 (PRISMA Flow Diagram).

### 3.4 Critique and synthesis of the literature

We critically analyzed and synthesized the selected literature, identifying both strengths and weaknesses in existing literature/conceptualizations. We assembled scattered elements of the appropriate approach to propose a path forward for understanding the intersection of the Metaverse and sustainable consumption.

Our methodology was guided by established literature review methodologies, as described by Van Wee and Bannister [20], ensuring rigor and comprehensiveness of our review. Our systematic literature review provided us with 59 articles of whom only 21 were directly related to understand the intersection of Metaverse and sustainable consumption.

### 3.5 Documents identified

In this paper, a general literature review was performed to identify studies that explored the role of Metaverse in promoting sustainable consumption behaviors. The search terms were chosen based on their relevance to the research question and objectives, and the databases used in the search included ScienceDirect and Scopus. Table 1 showcases the count of papers sourced from each database, along with the cumulative number of publications incorporated into the review. The papers discovered were subsequently screen by their titles and abstracts to exclude those that were not pertinent to the study.

### 3.6 Papers excluded

The titles and abstracts of the 21 identified articles were examined to identify potentially related papers. The search, employing relatively broad terms related to research methodologies and statistics, unsurprisingly led to a large number of irrelevant papers, given the general nature of the terms and their common use in methodological and analytical sections of empirical works. The exclusion of papers was based on the following criteria:

(a) Several publications that addressed the use of the Metaverse in educational techniques were omitted from this review, as they failed to provide either qualitative or quantitative assessments of the interventions' effectiveness [5,21,22].
(b) A number of papers pinpointed by the search criteria discussed the vast range of available Metaverses; however, these were excluded from the review because they lacked an empirical evaluation of the tool's impact over sustainability [[23], [24], [25]].
(c) Numerous documents found through the search terms discussed the Metaverse, virtual reality, augmented reality or mixed reality, but the paper was rejected if the platform did not directly over optimization of resources, social inclusion and overall sustainability concepts. Several publications describing Metaverse to support marketing activities were discovered, but their material was deemed insufficiently relevant with regards to sustainability and sustainable consumption [[26], [27], [28]].

### 3.7 Papers chosen

21 studies that reported on and assessed the Metaverse's connection to sustainability in terms of research methodologies and statistics were found to meet the inclusion standards. As a result, they were considered pertinent to the present investigation.

### 3.8 Classification of documents

As with earlier literature surveys in the field of Metaverse [29], the papers discovered in this review addressed a variety of subjects. It was helpful to categorize the 21 publications according to the type of impact it had over multiple implications for sustainability. They described: Metaverse and travel optimization in the tourism industry, Metaverse and education enhancement, Metaverse, marketing and sustainable consumption, Metaverse and life improvement thanks to smart cities and Metaverse and social inclusion. These categories and the quantity of publications covering each topic are explained below. A detailed overview of all 21 papers is provided in the tables in the Appendix, encompassing aspects like the authors, the methodology and structure of the research, the data sources from which the papers were derived, the objectives of the studies, the specific topics in research methods that were addressed, and the findings of each study.

## 4 Results

### 4.1 Metaverse: implications for urban ecosystems

The integration of the Metaverse with smart cities is a subject of extensive exploration in modern literature, uncovering technologies that promote sustainability in urban living. One significant innovation is digital twins (DT) technology, which creates virtual counterparts of real-world entities [30]. This technology aids in building simulation models to analyze real-world performance, resolving ‘what-if’ scenarios in urban planning, and leading to cost and time savings in modeling and testing [9].

Resource management is a vital aspect, especially in cities that consume a vast portion of the world's resources. The strain on resources like land, water, and minerals could increase without effective management [31,32]. The Metaverse could lead to an improvement in resource management by cutting down physical travel, although integrating virtual and physical realms requires extensive collaboration and innovation [7,33]. Consideration must also be given to governance structures within the Metaverse and to the synchronization of physical and virtual activities. Improving virtual urban experiences does not automatically translate to enhanced physical aspects [34].

Traditional urban planning models can inadvertently foster isolation by encouraging private car ownership and limiting accessible shared spaces [35]. However, the Metaverse offers alternatives to enhance social interactions and inclusivity. It provides a space where people can overcome barriers like distance and racial segregation, play, socialize, and explore together [9,36]. Virtual worlds might even become centers for corporate training and recruitment [37], though challenges such as ethical concerns, privacy issues, and accessibility barriers must be tackled [33,38,39].

The intersection of the Metaverse with tourism presents unique opportunities and challenges. The blending of physical and digital worlds can support sustainable tourism by offering virtual experiences of landscapes and attractions [40,41]. Although some experiences cannot be replicated virtually, the Metaverse can still encourage real-life visits and aid in conservation efforts. It holds implications for preserving endangered heritage sites, creating job opportunities, and supporting local businesses [42,43]. Examples include the use of a Metaverse social app in Santa Monica and blockchain technology's potential to conserve historical places.

Research in Thailand has also delved into Metaverse initiatives for virtual tourism attractions, demonstrating its potential to enable targeted tourist groups, train local content developers, and support tourism in the post-Covid-19 era [44]. The exploration of domestic and international tourists' perspectives on the extended Metaverse for tourism purposes is suggested for future research [44].

### 4.2 Metaverse and education

According to the reviewed literature, education is a significant area of knowledge that can benefit from the integration of the Metaverse. The papers emphasize the role of Generation Z, who are comfortable with online schooling and view the digital and real worlds as equally important, having grown up with technology [45]. The authors view the Metaverse as a massive framework with futuristic digital features such as interactivity, authenticity, and portability. In the realm of education, the Metaverse offers various advantages, such as low learning costs and risks [46], unlimited time and space, personalization, promotion of communication, and low cost.

One benefit is that students can learn about topics like Chemistry and Physics through real-world experiments in a safe environment, enabling them to better understand the material [46]. In addition, the Metaverse removes geographical and time constraints, allowing students to experience historical events and study environments from around the world without having to physically travel there [33]. The potential to connect students globally also fosters a greater understanding of sustainability issues and the importance of international collaboration [47].

The use of blockchain technology in the Metaverse also helps prevent academic misconduct (Meta, 2021). Another advantage is personalization, as students can create their own avatars using digital twin generators or simulators, which can boost their confidence and engagement in their education. The education system can also use personal data to create individualized content and course plans. Communication is also improved in the Metaverse, as teachers can organize meetings with their students in virtual rooms, and students can collaborate, study, and socialize with their peers. Distance hinders online classroom communication [48], but the use of avatars in the Metaverse allows everyone to see each other, share files, and play games, promoting student-teacher interactions, including classmates' friendships [48].

Furthermore, the Metaverse can contribute to the promotion of sustainable practices in education. For example, virtual field trips and conferences can reduce the need for physical travel, minimizing the environmental impact of educational activities [22].

The cost of education is also reduced with the use of the Internet of Things and AI search systems that can help students quickly gather and organize learning materials and reminders of relevant topics and resources. Teachers can also use big data and educational behavior tools to provide individualized assignments and help students become more independent learners. Finally, students can use their personal learning network to automatically gather and submit information related to their learning goals.

However, some challenges need to be addressed to ensure the effective integration of the Metaverse in education. For instance, access to technology and the digital divide can exacerbate existing inequalities in education [49]. Additionally, privacy concerns and the potential for misuse of personal information should be carefully considered when implementing Metaverse technologies in educational settings [21].

### 4.3 Metaverse and sustainable marketing

The examined literature reveals that the Metaverse holds vast potential for immersive and captivating brand experiences [50]. Several factors make the Metaverse particularly advantageous for corporate branding. Firstly, its three-dimensional environment allows companies to create lifelike simulations of their offerings, helping customers grasp what they are buying and letting businesses test novel product concepts before fully investing in them. Secondly, the Metaverse's ongoing existence enables brands to maintain their presence without continuous advertising, fostering client loyalty and community building. Lastly, the shared reality of the Metaverse facilitates closer connections between companies and customers, resulting in unique customer experiences and reinforced existing relationships [18].

The literature outlines how businesses can build a robust presence, engage users, and deliver value using marketing, branding, and advertising in the Metaverse. Furthermore, it underscores the importance of addressing the Metaverse's distinct aspects when devising marketing strategies [51]. Brands like Nike and Reebok illustrate the effective implementation of Metaverse marketing. Celebrities can also extend their brands in the Metaverse by creating avatars and interacting with users [52]. Nevertheless, businesses and celebrities must remain cautious about potential risks, such as avatar theft and negative user-generated content (Molina, 2021).

Ownership and paid advertising in the Metaverse pose challenges [53]. Conventional advertising features include 1) influence, 2) targeting specific audiences, 3) financial sponsorship, and 4) conveying advertising messages designed to trigger particular responses. The Metaverse's controlled environment, users' virtual identities, and distinctive features for fostering customer-brand interactions may impact how consumers perceive marketing messages [54].

Literature on the Metaverse and sustainable marketing highlights the necessity of engaging with users in their digital forms. Advertising may need to prioritize delivering memorable and enjoyable experiences over promoting products. Marketers should create non-intrusive ads that offer users enough information to make informed choices [45]. While interactive ads might attract Metaverse users, the platform's infancy means marketing and promotion strategies in this digital domain remain uncertain [25].

Additional studies emphasize the potential drawbacks of not adopting the Metaverse for promotional strategies and brand enhancement, as this oversight may result in a decrease in visibility, engagement, and client acquisition [38]. Companies that do not capitalize on the Metaverse may lag behind competitors who do [37]. By establishing a presence in the Metaverse, businesses can tap into a global customer base that spends more time in virtual worlds [2].

### 4.4 Bibliometric analysis

**Fig. 2:**
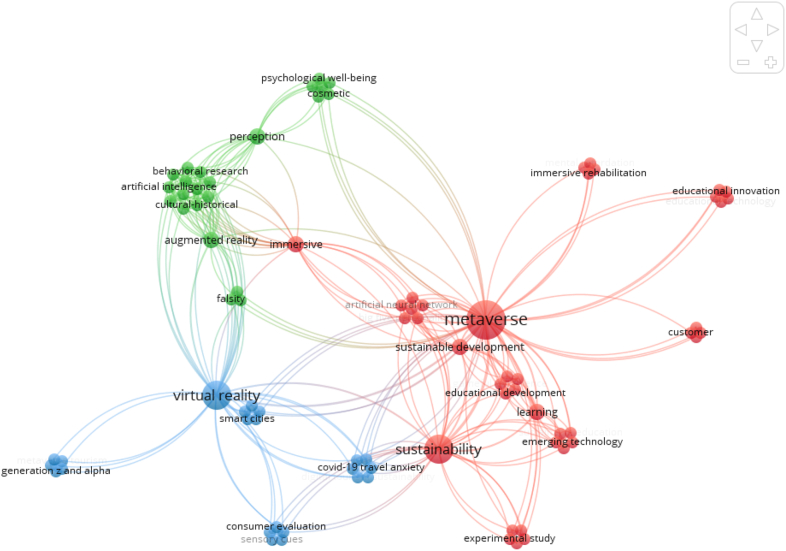
Co-occurrence analysis.

We conducted a keyword co-occurrence analysis to understand the prevalent research themes within our database and discover the frequency with which specific keywords appear together, focusing on the relationships between co-occurring terms [55]. Using VOSviewer, we performed this analysis to identify and evaluate the emerging trends in the Metaverse and sustainable consumption. The findings, as shown in Fig. 2, reveal the most frequent keywords, with ‘Metaverse’ being the most common (88 instances), followed by ‘virtual reality’ (49 instances), ‘Sustainability’ (45 instances), and ‘emerging technologies' (15 instances). The keywords were categorized into three main clusters, each representing a central theme. The green cluster centers on ‘Emerging Technologies and Human Behavior,’ the red cluster emphasizes Digital Solutions for Sustainable Development and Education, and the blue cluster relates to Virtual Reality Applications for Smart Cities. These results provide significant insights into the intersections between the Metaverse and domains closely related to sustainable consumption, highlighting the substantial amount of research that has been conducted in these areas.

The exploration of smart cities within the Metaverse has focused on leveraging digital twin technology and data-driven simulations to optimize resource management, improve urban planning, and reduce environmental impact. By integrating the Metaverse with smart city initiatives, it is possible to create more sustainable urban environments that promote efficient resource utilization, sustainable transportation, and enhanced quality of life.

Virtual reality and augmented reality technologies have been studied in relation to sustainable development, particularly in the context of education, immersive rehabilitation, and behavioral research. These technologies offer innovative ways to educate and engage individuals in sustainable practices, simulate real-world scenarios for behavior change interventions, and provide immersive experiences for rehabilitation and therapy purposes. By utilizing virtual reality and augmented reality in these areas, it becomes possible to promote sustainable behaviors, enhance learning outcomes, and improve individual well-being.

The application of artificial intelligence (AI) in the Metaverse has implications for sustainable consumption by enabling personalized recommendations, optimizing resource allocation, and supporting decision-making processes. AI algorithms can analyze user data and preferences to provide tailored suggestions for sustainable products and services, thereby influencing consumer behavior towards more eco-friendly choices. Additionally, AI-powered systems can optimize energy usage, reduce waste, and enhance operational efficiency, contributing to sustainability efforts in various sectors.

The integration of the Metaverse with sustainable development goals and educational innovation has opened up opportunities for immersive learning experiences, global collaboration, and the dissemination of sustainable practices. Through virtual environments, individuals can explore and understand complex sustainability challenges, engage in virtual collaborations for sustainable projects, and access educational resources from diverse perspectives. This fosters a deeper understanding of sustainability issues and encourages the adoption of sustainable consumption behaviors.

Lastly, the Metaverse has been examined in the context of addressing travel anxiety during the COVID-19 pandemic. By providing virtual travel experiences and remote collaboration platforms, the Metaverse offers alternatives to physical travel, reducing carbon emissions associated with transportation and alleviating travel-related stress and anxiety. This promotes sustainable travel behaviors and encourages individuals to consider virtual options as viable alternatives for business meetings, conferences, and leisure activities.

Overall, the exploration of the Metaverse in conjunction with these domains demonstrates the potential for technology-driven solutions to foster sustainable consumption behaviors, optimize resource utilization, promote education and awareness, and address challenges related to travel and mobility. The interplay between Metaverse and these domains offers new avenues for sustainable development and contributes to the ongoing discourse on achieving a more sustainable future.

From the analysis of highly cited documents below, several key insights emerge regarding the intersection of the Metaverse and sustainable consumption. The interdisciplinary nature of the research is evident, with contributions from fields such as computer science, psychology, education, urban planning, and sustainability. For instance, Choi et al. [56] explore the building of a Korean DMZ Metaverse using a web-based platform, highlighting the potential for immersive virtual environments to support sustainable consumption practices. Golf-Papez et al. [57] discuss the concept of synthetic customer experiences in the Metaverse, emphasizing the need for responsible engagement and the implications for sustainable consumption. Zhou et al. [58] focus on self-powered sensing technologies for human Metaverse interfacing, shedding light on the potential of innovative technologies to facilitate sustainable interactions within virtual communities. Arpaci et al. [6] investigate the social sustainability of the Metaverse, employing a hybrid SEM-ANN approach to understand the role of user traits and technology acceptance in shaping sustainable consumption behavior. Lee et al. [59] examine the application of technology-enhanced education through Metaverse-linking and VR-making, highlighting the role of immersive learning experiences in fostering sustainable learning practices. These highly cited papers, along with others such as Valaskova et al. [60], Allam et al. [7], Park et al. [8], Garrido-Iñigo et al. [61], and Cheung et al. [62], provide valuable insights into the field, guiding scholars and professionals who are keen to investigate the implications of the Metaverse on sustainable consumption.

## 5 Discussion

This section discusses the implications for the future of the Metaverse on Sustainability that emerged from the review, addressing both the positive and potential negative effects with a deeper integration of associated literature on topics such as Information and Communication Technology (ICT), Artificial Intelligence (AI), social media and internet-related issues.

### 5.1 Metaverse potential implications to model and predict the future

Drawing on studies on ICT and AI's potential in predictive modeling, we find parallels with the Metaverse's capabilities to enhance understanding and prediction of sustainability-related outcomes [63]. Although the Metaverse offers unique opportunities for simulating future scenarios and evaluating the potential environmental impacts of different projects, it is essential to remember that these simulations are dependent on the quality of the input data and the underlying models, a challenge that is well-documented in the AI field [64]. Thus, caution is needed when interpreting these simulations' results, and continuous refinement and validation against real-world data is essential.

### 5.2 Metaverse's implications to decrease expenditures

The potential for the Metaverse to enable people to live, work, and consume in virtual environments without the need for physical relocation indeed mirrors benefits observed in the transition to digital services in various sectors. This opens opportunities for individuals to reduce their carbon footprints by reducing the need for travel, and to have access to a wider variety of products and services without having to physically go to a store [11]. However, like the internet, this transition could also intensify energy use and carbon emissions (References). Furthermore, as observed in the social media landscape, a shift towards virtual consumption could potentially lead to new forms of consumption and waste, such as electronic waste or ‘e-waste’ [65]. [66]. [67].

It is important to consider the potential consequences of virtual consumption and how it may impact the environment. While virtual environments can be designed to be more sustainable than physical environments, it is not yet clear how they will affect energy use and carbon emissions [68]. Additionally, the shift towards virtual consumption may lead to a decrease in the demand for physical goods, which could have unintended consequences for industries that rely on the manufacture and sale of physical products [29]. Whether there will be equal or higher demand for similar virtual products in the Metaverse remains to be seen. To fully understand the implications of virtual consumption in the Metaverse, it will be important to gather data and conduct research on the subject [69]. This can be done through studies of existing virtual environments, as well as through the creation of new virtual environments that are designed with sustainability in mind (Kozinets, 2023). Additionally, the policies and regulations put in place by the governing bodies of the Metaverse will play a critical role in shaping the future of virtual consumption and sustainability [70].

The potential for virtual consumption in the Metaverse to drive sustainable consumption patterns is an exciting possibility, but much remains to be seen about whether this will become a reality. Further research is needed to fully understand the implications of virtual consumption for the environment, and the role that policies and regulations will play in shaping its future.

### 5.3 Metaverse's implications to improve areas such as education, marketing, urban sustainability

An area where virtual consumption in the Metaverse has the potential to drive sustainable consumption patterns is education. The Metaverse allows for more accessible and affordable education by reducing the need for physical travel to educational institutions [71]. Additionally, the ability to access a wider range of educational resources in virtual environments opens opportunities for individuals to continue their education without having to physically move to a new location [72].

In the higher education sector, the Metaverse's virtual landscapes can foster a unique learning environment, which can lead to improved retention of information and a deeper understanding of subject matter [48]. This, in turn, can result in a decrease in the need for physical textbooks, which can reduce the environmental impact of the educational sector. However, as with all aspects of virtual consumption in the Metaverse, it is essential to understand the potential consequences for the environment. The increased use of technology for education in virtual environments could result in a rise in carbon emissions and power consumption [73]. Additionally, the unequal distribution of access to technology may create disparities in access to education in virtual environments, which could exacerbate existing inequities in the educational system [49].

It is crucial for policymakers and educators to carefully consider the sustainability implications of virtual consumption in education and to put in place policies and practices that ensure access to technology and virtual environments is equitable and sustainable. Virtual consumption in the Metaverse has the potential to drive sustainable consumption patterns in the area of education by reducing the need for physical travel, providing more accessible and affordable education, and improving the quality of education using virtual environments. However, it is important to consider the potential consequences for the environment and to put in place policies and practices that ensure access to technology and virtual environments is equitable and sustainable.

The marketing industry could also be influenced in various ways by virtual consumption within the Metaverse. One of the key benefits of virtual environments is that they provide a unique opportunity for companies to engage with their target audience in more immersive and realistic environments [74]. This can lead to increased brand awareness and a deeper connection with customers, which can result in increased sales and revenue [75]. Virtual environments provide new opportunities for companies to advertise their products through more engaging experiences, which can help to reduce the environmental impact of traditional marketing methods such as print advertising and physical product displays [76]. For example, companies can create virtual product displays in the Metaverse that enable customers to engage with their products in ways that were previously impossible [77].

It is important to note that virtual environments may bring both negative and positive effects to the forefront. On one hand, they might contribute to overconsumption and a culture of disposability, as customers may be more likely to purchase virtual goods and experiences that they would not consider buying in physical reality [78]. This can have harmful effects on the environment, as virtual goods and experiences require energy and resources to produce, even if they do not have a physical form [79].

Conversely, consumption within the Metaverse holds the potential to produce positive effects on urban sustainability. One of the key benefits of the Metaverse is that it can reduce the need for physical travel, which can have a significant impact on urban sustainability [7]. For instance, the Metaverse can offer opportunities for remote work and telecommuting, which can reduce the number of cars on the road and help to mitigate the environmental impact of urban transportation [56]. In conjunction with the initiatives to promote travel destinations via Metaverse tourism, this may also enable cities to promote their local businesses to potential investors, without the need necessarily for investors to expend resources for physically travelling to the location.

Additionally, the Metaverse can help urban planners and policymakers to simulate and test new urban design and planning strategies, which can help to promote sustainable urban development [80]. The Metaverse can represent a platform for urban planners to assess new green infrastructure and sustainability initiatives, which can help to identify areas for improvement and reduce the environmental impact of urban development [81].

However, it is important to note that virtual environments can also contribute to the sprawl of urban development and the consumption of resources and energy, as virtual spaces require energy and resources to maintain and operate [82]. This can have a negative impact on urban sustainability, as it can increase the overall consumption of resources and energy and contribute to the degradation of the environment [25].

### 5.4 Potential pitfalls or challenges to address

One current issue with the literature is with the ambiguous objectives related to the Metaverse. While some studies argued about the utility of Metaverse for helping people and students better understand complex sustainability issues [8], several other studies seemed very fixated on how the Metaverse could connect workers in virtual environments. It should be pointed out that although people can connect through a virtual world, this does not necessarily create substantive connections that make the Metaverse a better alternative to remote work on applications such as Zoom or Microsoft Teams. Moreover, current iterations of the virtual reality Metaverse have caused physical ailments such as dizziness or headaches, and it may not be viable for the Metaverse to require headsets.

Another challenge that should be addressed is whether the Metaverse will create an environment that contributes to better overall health of users. Technology addiction has still been widely studied and is still a significant issue. There have also been reported issues of sexual harassment and bullying happening in these Metaverse worlds, with some users arguing that they can commit these actions because it is in a virtual space [83]. How the Metaverse can regulate and prevent this problematic behavior while enabling people to find work and educational opportunities remotely should be studied further in order to determine if the Metaverse can truly help realize a more sustainable future.

## 6 Future directions for research, legal challenges and opportunities

Most of the reviewed papers in this study are in the field of business, with few addressing the Metaverse and sustainability specifically. This highlights a gap in the literature and an opportunity for future research. Legal scholars could also explore the regulatory framework governing the Metaverse, including intellectual property rights, privacy laws, and accessibility standards. The legal landscape surrounding the Metaverse is complex and still in its formative stages, presenting a fertile ground for future research. Key areas include the conflict of states for control over transboundary virtual spaces, requiring the examination of jurisdiction and potential international regulation. Legal scholars may also explore the status and legitimacy of virtual transactions, the legal implications of cryptocurrencies, and the procedures for mutual settlements within the Metaverse. In addition to intellectual property rights, privacy laws, and accessibility standards, the law will have to adapt to new realities such as virtual property rights, data security, content regulation, and virtual financial transactions. International efforts, such as the European Union's exploration of regulations for the Metaverse, underline the global relevance of these legal challenges. The rapid development of the Metaverse calls for agile and insightful legal research, ensuring that laws and regulations evolve cohesively with the technological landscape, safeguarding users' rights, and promoting ethical, sustainable virtual environments.

Furthermore, studies could explore the Metaverse's potential to promote sustainable consumption patterns, examining the legal implications of virtual goods and experiences replacing physical products. The development of the Metaverse could be analyzed in the context of sustainable urban planning and design, considering legal aspects such as zoning laws and transportation regulations. The role of policy and regulation in shaping the sustainability outcomes of the Metaverse should be considered, investigating governments' potential to set sustainability standards for virtual environments and ensure equitable access to technology.

In order to properly evaluate the impact of the Metaverse on sustainability, research must empirically investigate its effects. This includes legal considerations, such as how Metaverse technologies comply with existing laws and how they might influence future legislation. Research has already highlighted potential pitfalls of the Metaverse, including legal challenges related to bullying, harassment, and security issues. These areas warrant further legal analysis.

Accessibility and opportunity within the Metaverse are also key issues. Legal research could explore how regulations ensure that the Metaverse is accessible to all and that opportunities, such as new job prospects or virtual travel, are governed by fair and transparent rules.

In order to properly evaluate the impact of the Metaverse on sustainability, much more research must be done that does not merely describe the application of the Metaverse to various sustainability-related disciplines, but empirically investigates its impacts to these different areas. Quite a few articles discussed the potential of the Metaverse to help with modelling and predicting future outcomes [7,34]. How Metaverse technologies can help business or government leaders make better decisions can help not only show the efficacy of the Metaverse for achieving sustainability but provide further ideas on how the Metaverse should be utilized to address many of the complex problems within sustainability.

Research has already highlighted potential pitfalls of the Metaverse. As the Metaverse shares similarities with other online environments, there have already been reported issues of bullying, harassment, and security issues [75]. Whether the Metaverse can become a better environment that overcomes these issues is worth investigating further in the future.

Another issue to further consider is the issue of accessibility and opportunity within the Metaverse. While scholars have noted the potential of the Metaverse to address social inequalities in the world, there is a huge number of resources required to develop the Metaverse. How the Metaverse environments can be made readily accessible for everyone will be a key issue to explore further. It should also be emphasized that accessibility is not the only key issue. The opportunities and benefits that users can receive upon entering these Metaverse environments, whether it is new job opportunities [56] or opportunities to travel to a new destination that would otherwise be inaccessible [44], should also be documented and studied further. How the Metaverse can offer these opportunities, as well as how different individuals utilize these opportunities, will be a key area to explore further and will shed new insight on the impacts of the Metaverse.

Moreover, despite the number of education-related Metaverse interventions that are now in development, there is still a lack of evidence that Metaverse interventions will create better learning outcomes for students. More experimental studies that compare the use of Metaverse environments to more traditional learning environments would help highlight the effectiveness of the Metaverse for learning.

In conclusion, the Metaverse has already offered interesting possibilities for addressing various issues of sustainability. However, for the Metaverse to achieve its potential and avoid pitfalls of other platforms, it must develop in close coordination between various stakeholders, and seek to develop equal opportunity and accessibility among individuals of all social classes and backgrounds. This will require much more dedicated empirical research that goes beyond merely describing the Metaverse for a more sustainable world and analyze the substantive impact the Metaverse has on making sustainability a reality.

## 7 Conclusion

Our exploration of the potential applications of the Metaverse in sustainable consumption reveals several key insights. Firstly, we highlight the potential applications of the Metaverse as a powerful tool for sustainable consumption, particularly in promoting non-plastic alternatives. In emerging markets such as India, the Metaverse can play a significant role in raising awareness and influencing behavioral change towards more environmentally-friendly consumption habits. This aligns with objectives of campaigns like Sabka Saath Sabka Vikas (Collective Effort, Inclusive Development) and Swachh Bharat Abhigyan (Clean India Mission), both of which have spurred sustainable behaviors in these markets [84].

Our research also reveals that digital nudges or gamification techniques within the Metaverse could be instrumental in promoting sustainable behaviors. This finding signal exciting opportunities for future exploration in the intersection of technology, consumer behavior, and sustainable consumption.

Additionally, we have assessed the potential economic and environmental impacts of the Metaverse in relation to sustainable consumption. Subsequent studies might probe further into this area, focusing on life cycle assessments of virtual platforms and their environmental impacts, and exploring how the Metaverse can contribute to more sustainable economic models.

### 7.1 Implications for future research

This review has highlighted several areas that warrant further exploration. Future research could focus on developing public policy recommendations for the integration of sustainable consumption into the Metaverse, examining the impact of different types of digital nudges or gamification techniques on sustainable behaviors, and conducting a more comprehensive understanding of the economic and environmental impacts of the Metaverse on sustainable consumption.

We encourage researchers and policymakers to collaborate on the development of guidelines and regulations for the responsible use of the Metaverse, ensuring that its growth aligns with sustainability and social equity objectives. This collaboration could help bridge the gap between technological advancements and sustainability objectives, leading to innovative solutions that promote economic growth and environmental conservation. As we continue to assess the potential of the Metaverse, it is important to question our assumptions and challenge our biases to build a platform that is inclusive, equitable, and sustainable.

This review paper not only identifies the current domains of literature in the field of Metaverse and sustainable consumption but also highlights the potential of the Metaverse as a platform for modeling and predicting the future. The findings suggest that further research is needed to fully explore the potential applications of the Metaverse in sustainable issues, particularly in understanding the economic and environmental impacts of the Metaverse on sustainable consumption. Overall, this review paper contributes to the emerging field of Metaverse and sustainable consumption and provides valuable insights into the opportunities and challenges for creating a more sustainable future using advanced digital technologies.

To address the policy implications and proposals for future research, this review identifies several avenues to explore. One area of investigation could be the development of public policy recommendations for the integration of sustainable consumption into the Metaverse. This may involve exploring how virtual platforms can be designed to encourage sustainable behaviors among users and how sustainable practices can be integrated into virtual marketplaces.

Another area for future research is the examination of consumer behavior and motivations in the Metaverse, as well as the role of technology in promoting sustainable consumption. This could involve investigating how different types of digital nudges or gamification techniques can be used to encourage sustainable behaviors in virtual environments. It could also involve exploring the policy and regulatory frameworks governing these interventions, as well as evaluating the long-term efficacy and potential unintended consequences of employing such methods to foster sustainable practices within the Metaverse.

Additionally, future research could focus on a more comprehensive understanding of the economic and environmental impacts of the Metaverse on sustainable consumption. This could involve conducting life cycle assessments of virtual platforms to understand their environmental impacts, as well as exploring how the Metaverse can contribute to more sustainable economic models, such as the circular economy.

Ultimately, the Metaverse presents a unique opportunity to explore new frontiers in human creativity and innovation, while also addressing some of the most pressing challenges facing our planet. As George, Fernando, George, Baskar, and Pandey [85] point out, the Metaverse offers a platform for experimenting with new ways of living, working, and consuming that are less harmful to the environment and more equitable for all. Additionally, the Metaverse may allow us to transcend our current limitations and unlock new possibilities for personal growth and development [52]. However, as we explore these new frontiers, we must also recognize the importance of building a sustainable infrastructure that can support the growth of the Metaverse without sacrificing our natural resources [10]). Moreover, we must be willing to question our assumptions and challenge our biases in order to build a Metaverse that is inclusive, equitable, and sustainable [11]. By harnessing the power of the Metaverse for good, we can create a world that is more sustainable, more equitable, and more inspiring than anything we have seen before. In conclusion, this review paper has assessed the potential applications of the Metaverse in various areas related to sustainable consumption, highlighting the need for further research to explore its potential benefits and challenges. By focusing on areas such as policy implications, consumer behavior, and the economic and environmental impacts of the Metaverse, future research can contribute to a better understanding of how this emerging technology can be harnessed for sustainable development.

Researchers and policymakers are encouraged to collaborate in the development of guidelines and regulations for the responsible use of the Metaverse, ensuring that its growth is aligned with the goals of sustainability and social equity. Additionally, interdisciplinary research can help bridge the gap between technological advancements and sustainability objectives, leading to the development of innovative solutions that promote both economic growth and environmental conservation.

## Author contribution statement

All authors listed have significantly contributed to the development and the writing of this article. </p>

## Data availability statement

Data will be made available on request.

## Declaration of competing interest

The authors declare that they have no known competing financial interests or personal relationships that could have appeared to influence the work reported in this paper.

## Figures and Tables

**Table 4:** Papers included in the analysis on Metaverse and Smart Cities

Title	Research approach, study design and aim	Authors	Results	
Understanding the social sustainability of the Metaverse by integrating UTAUT2 and big five personality traits: A hybrid SEM-ANN approach	Quantitative	Arpaci, I., Karatas, K., Kusci, I., & Al-Emran, M. (2022)	In the context of the Metaverse's social sustainability, significant factors such as performance expectancy, social influence, hedonic motivation, price value, habit, agreeableness, neuroticism, and openness play a crucial role. On the other hand, elements like effort expectancy, facilitating conditions, conscientiousness, and extraversion don't exhibit a notable impact. Together, these factors account for 80% of the variance in social sustainability. Furthermore, habit emerges as the most critical predictor of social sustainability, as evidenced by the ANN analysis.	
Construction 4.0, Industry 4.0, and Building Information Modeling (BIM) for Sustainable Building Development within the Smart City	Review	Chen, Y., Huang, D., Liu, Z., Osmani, M., & Demian, P. (2022)	With the utilization of VOSviewer software for keyword-clustering analysis and application, the top 11 most productive subject areas have been discerned. In addition, considering the whole-building life cycle aids in uncovering research gaps and trends, thereby offering guidance and recommendations for future investigations.	
The Metaverse as a Virtual Form of Smart Cities: Opportunities and Challenges for Environmental, Economic, and Social Sustainability in Urban Futures	Review	Allam, Z., Sharifi, A., Bibri, S. E., Jones, D. S., & Krogstie, J. (2022)	This research has the potential to assist urban policymakers in gaining a clearer comprehension of the Metaverse's effects on tech-mediated practices and urban initiatives. It helps in evaluating both the advantages and drawbacks of this techno-urban vision.	
Working in the Metaverse: Does Telework in a Metaverse Office Have the Potential to Reduce Population Pressure in Megacities? Evidence from Young Adults in Seoul, South Korea	Case study	Choi, H. Y. (2022)	The research illustrates the varying effects of different forms of telework in alleviating population stress in major cities, offering actionable guidance for policymakers and strategists to endorse and facilitate this approach.	

**Table 5:** Papers included in the analysis on Metaverse and Tourism

Title	Research approach, study design and aim	Authors	Results
Building Korean DMZ Metaverse Using a Web-Based Metaverse Platform	Application Case	Choi, S., Yoon, K., Kim, M., Yoo, J., Lee, B., Song, I., & Woo, J. (2022)	This text outlines the specific design characteristics, architectural framework, models, and functionalities of OnTwins, a Metaverse platform adaptable to the Korean government's IT operational environment. Additionally, the text introduces the DMZ Metaverse public service, which is constructed on this platform. A comparison with other Metaverse platforms is provided, followed by a discussion of potential directions for future research.
Meet Your Digital Twin in Space? Profiling International Expat's Readiness for Metaverse Space Travel, Tech-Savviness, COVID-19 Travel Anxiety, and Travel Fear of Missing Out	Quantitative	Zaman, U., Koo, I., Abbasi, S., Raza, S. H., & Qureshi, M. G. (2022)	The readiness of international expatriates for Metaverse space travel is found to be positively affected by their anxiety about COVID-19-related travel. Additionally, those expats who display strong tendencies toward travel FOMO and technological adeptness are more likely to see their COVID-19 travel anxiety exert a greater influence on their willingness to engage in Metaverse space travel.
Metaverse tourism for sustainable tourism development: tourism agenda 2030	Review	Go, H., & Kang, M. (2022)	Metaverse-related products and experiences have the potential to enhance the diversity of tourism options, aligning with sustainable tourism goals. By creating licensed and profitable Metaverse tourism offerings, destinations could see increased profitability. Such developments should be in line with the UNWTO's Sustainable Development Goals (SDGs).
Smart hospitality: from smart cities and smart tourism towards agile business ecosystems in networked destinations	Review	Buhalis, D., O'Connor, P., & Leung, R. (2022)	The study explores the concept of smart hospitality, leveraging the principles of smart cities and smart tourism to create flexible business ecosystems within connected destinations. By examining current literature, the researchers crafted a conceptual framework and presented an extensive agenda for future research in this area.
Intelligent cathedrals: Using augmented reality, virtual reality, and artificial intelligence to provide an intense cultural, historical, and religious visitor experience	Multiple case study	Allal-Chérif, O. (2022)	Immersive technologies offer the potential to conserve cathedrals and enable individuals to explore otherwise inaccessible areas from their homes. These virtual explorations can cater to personal desires, even those considered impossible or forbidden, culminating in profound spiritual experiences. The perception of these virtual encounters can be as compelling and significant as real-life experiences.

**Table 6:** Papers included in the analysis on Metaverse and Education

Title	Research approach, study design and aim	Authors	Results
Technology-Enhanced Education through VR-Making and Metaverse-Linking to Foster Teacher Readiness and Sustainable Learning	Qualitative	Lee, H., & Hwang, Y. (2022)	The transformative experiences of Virtual Reality Making (VRM) for educational content are instrumental in enhancing pre-service teachers' technological readiness and fostering the 4Cs (Critical Thinking, Creativity, Collaboration, Communication) in digital citizenship. Additionally, these experiences contribute to the perception of pedagogical benefits.
Identifying World Types to Deliver Gameful Experiences for Sustainable Learning in the Metaverse	Qualitative	Park, S., & Kim, S. (2022)	The findings of this research have the potential to foster equal educational opportunities for learners by facilitating the development of innovative educational environments. This advancement aligns with the pursuit of achieving the fourth sustainable development goal.
The reality of virtual worlds: pros and cons of their application to foreign language teaching	Mixed methods	Garrido-Iñigo, P., & Rodríguez-Moreno, F. (2015)	The paper outlines an experiment using OpenSim as a means to teach French to tourism students at Universidad Rey Juan Carlos. It delves into the advantages of employing virtual worlds in education, detailing the instructional objectives and techniques used. Additionally, the authors evaluate the language acquisition outcomes and contemplate the interplay between technology and pedagogy in education within virtual environments.
Design and validation of a questionnaire for the evaluation of educational experiences in the Metaverse in Spanish students (METAEDU)	Quantitative	López-Belmonte, J., Pozo-Sánchez, S., Lampropoulos, G., & Moreno-Guerrero, A. J. (2022)	The research focused on creating an assessment tool for educational experiences within the Metaverse. A total of 362 Spanish students took part in the study, and the tool's validity and reliability were established through content validity, expert judgment, construct validity, and reliability tests. The findings confirm that the instrument is a credible and consistent means for evaluating education in the Metaverse.

**Table 7:** Papers included in the analysis on Metaverse and Social Sustainability

Title	Research approach, study design and aim	Authors	Results
The significant transformation of life into health and beauty in Metaverse era	Review	Lee, J., & Kwon, K. H. (2022)	Study analyzed changes in health and beauty perceptions among cosmetic consumers in the WC era in the Metaverse. 32 papers were researched and found that a new platform is needed to reflect the changing needs of consumers in the beauty market. The study concludes that a new platform is needed to integrate health and beauty in the WC era.
Virtual reality based multiple life skill training for intellectual disability: A multicenter randomized controlled trial	Quantitative	Cheung, J. C. W., Ni, M., Tam, A. Y. C., Chan, T. T. C., Cheung, A. K. Y., Tsang, O. Y. H., … & Wong, D. W. C. (2022).	This study tested a VR-based life skill training program for intellectually disabled individuals. Results showed VR had greater improvement in cooking and cleaning, while traditional training improved shopping, cooking, and cleaning tasks. VR also showed better memory improvement.

**Table 8:** Papers included in the analysis on Metaverse and Sustainable Marketing

Title	Research approach, study design and aim	Authors	Results
Virtual is so real! Consumers' evaluation of product packaging in virtual reality	Quantitative	Branca, G., Resciniti, R., & Loureiro, S. M. C. (2022)	The study investigates consumer behavior in virtual reality (VR) concerning the evaluation of packaged products, specifically focusing on manipulating packaging cues. The results indicate that consumer behavior in VR closely aligns with behavior in everyday life, with only minor differences. The study also demonstrates that VR is an effective research environment for examining sensory cues and intangible attributes. The findings suggest that business managers can utilize VR for product and packaging development, facilitating a more sustainable process.
Virtual Marketplace Dynamics Data, Spatial Analytics, and Customer Engagement Tools in a Real-Time Interoperable Decentralized Metaverse	Review	Valaskova, K., Machova, V., & Lewis, E. (2022)	The article focuses on the lack of research on virtual marketplaces in the Metaverse. The authors conduct a literature review of 83 articles and find that tailored product data and personalization through machine learning predictions can lead to customer engagement. The study contributes to the literature on sustainable businesses in the Metaverse.
Embracing falsity through the Metaverse: The case of synthetic customer experiences	Conceptual	Golf-Papez, M., Heller, J., Hilken, T., Chylinski, M., de Ruyter, K., Keeling, D. I., & Mahr, D. (2022).	Businesses face challenges in the Metaverse market. Success depends on viewing it as a unified reality and harnessing the promise of falsity. The authors offer examples of how to succeed in this new reality.
The PSICHE framework for sustainable consumption and future research directions	Review	Nascimento, J., & Loureiro, S. M. C. (2022)	Article to understand the key factors influencing environmentally sustainable consumer choices by examining 161 articles in the Web of Science and Scopus databases. The study found nine thematic clusters and created the PSICHE framework to predict green purchasing behaviors, including product-related factors, social influences
Self-powered sensing technologies for human Metaverse interfacing	Review	Zhou, Y., Xiao, X., Chen, G., Zhao, X., & Chen, J. (2022)	The article discusses self-powered sensing technologies for human interface in the Metaverse. The article highlights the need for self-powered sensing technologies to enhance user experience in the virtual reality world. The article suggests that further research is required to fully realize the potential of self-powered sensing technologies for human-Metaverse interfacing.
